# Cryoballoon ablation for paroxysmal atrial fibrillation in Japan: 2-year safety and efficacy results from the Cryo AF Global Registry

**DOI:** 10.1007/s10840-022-01132-0

**Published:** 2022-02-04

**Authors:** Masaomi Kimura, Atsushi Kobori, Junichi Nitta, Kenzo Hirao, Satoshi Shizuta, Takashi Kurita, Kaoru Okishige, Koichiro Kumagai, Junjiro Koyama, Kenichi Hiroshima, Osamu Inaba, Masahiko Goya, Yasuteru Yamauchi, Fred J. Kueffer, Daniel Becker, Ken Okumura

**Affiliations:** 1 Hirosaki University, Aomori, Japan; 2 Kobe City Medical Center General Hospital, Hyogo, Japan; 3 Sakakibara Heart Institute, Tokyo, Japan; 4 Medical Hospital, Tokyo Medical and Dental University, Tokyo, Japan; 5 Kyoto University Hospital, Kyoto, Japan; 6 Kindai University Hospital, Osaka, Japan; 7 Yokohama Minato Heart Clinic, Kanagawa, Japan; 8 Fukuoka Sanno Hospital, Fukuoka, Japan; 9 Saiseikai Kumamoto Hospital, Kumamoto, Japan; 10 Kokura Kinen Hospital, Fukuoka, Japan; 11 Saitama Red Cross Hospital, Saitama, Japan; 12 Yokohama City Minato Red Cross Hospital, Kanagawa, Japan; 13 Medtronic Inc, Minneapolis, USA; 14 Medtronic GmbH, Meerbusch, Germany

**Keywords:** Atrial fibrillation, Catheter ablation, Cryoballoon, Pulmonary vein isolation, Antiarrhythmic drug

## Abstract

**Purpose:**

Catheter ablation is a recommended rhythm control therapy after failed or intolerant antiarrhythmic drug (AAD) treatment for patients with atrial fibrillation (AF). This study evaluates clinical performance and safety of pulmonary vein isolation (PVI) using the cryoballoon (Arctic Front Advance) in Japan.

**Methods:**

*Cryo AF Global Registry* is a prospective, multi-center registry. Patients with paroxysmal AF (PAF) were treated at 10 Japanese hospitals. Efficacy was evaluated by freedom from a ≥ 30-s recurrence of AF/atrial flutter (AFL)/atrial tachycardia (AT), AF-related symptoms, and quality of life using the EQ-5D-3L questionnaire. The safety endpoint was serious device- and procedure-related adverse events.

**Results:**

The study included 352 patients with PAF (65 ± 10 years of age, 36% female, 36% without prior failure of AAD). Mean duration since first diagnosis of AF was 3.0 ± 5.5 years. Serious device- and procedure-related adverse event rate was 2.6% (95% *CI*: 1.2–4.8%). Freedom from AF/AFL/AT was 88.5% (95% *CI*: 84.7–91.4%) at 12 months and 86.7% (95% *CI*: 81.1–90.8%) at 24 months. The number of patients with ≥ 1 AF symptom was significantly decreased from 88% at enrollment to 22% (*p* < 0.01) at 12-month follow-up. General quality of life using EQ-5D did not improve significantly after 12 months in the summary score. However, in the visual analog scale score, there was improvement (5.8 ± 18.4; *p* < 0.01).

**Conclusions:**

This study demonstrates that cryoablation used for PVI is a safe and effective treatment in real-world use for patients with PAF in Japan.

**Supplementary Information:**

The online version contains supplementary material available at 10.1007/s10840-022-01132-0.

## Introduction

The Arctic Front Advance (Medtronic, Inc.) cryoballoon ablation system was first approved for the treatment of patients with antiarrhythmic drug (AAD)-refractory, recurrent, symptomatic, paroxysmal atrial fibrillation (AF) in Japan in 2014. Subsequently, the Cryo-Japan Post-Market Surveillance (PMS) study confirmed the short-term efficacy and safety of the cryoablation system for the treatment of patients with paroxysmal AF (PAF) in routine clinical practice [1]. As noted in the recent report from the Japanese Catheter Ablation Registry, AF catheter ablation accounted for nearly two-thirds of cardiac ablation procedures in 2018. Yet, reports of mid- and long-term outcomes of cryoballoon ablation for the treatment of patients with AF in Japan are limited [2]. In this report from the *Cryo AF Global Registry*, real-world efficacy, safety, and quality of life (QoL) outcomes in AF patients treated with the cryoballoon catheter in Japan are reported.

## Methods

### Study design

The *Cryo Global Registry* (ClinicalTrials.gov registration: NCT02752737) is an ongoing prospective, multi-center, observational, post-market study. The objectives of this sub-analysis were to evaluate acute procedural characteristics, safety, and efficacy when using the cryoballoon ablation catheter to treat patients with AF in Japan. Data were collected at ten hospitals in Japan (Supplemental Table S1). The study was conducted according to Good Clinical Practices (in compliance with local regulations and in accordance with the principles outlined in the Declaration of Helsinki). Each site received approval by an independent ethics/institutional review board and obtained written informed patient consent for all patients prior to enrollment. This study was sponsored by Medtronic, Inc.

### Patient population

All patients ≥ 18 years old with a planned procedure using the Medtronic cryoablation system were eligible for inclusion in the registry. The present examination includes patients with PAF that were enrolled and treated at study centers in Japan for initial catheter ablation of AF. Patients were classified as having PAF if they had an episode(s) of AF that terminated spontaneously or with intervention within 7 days of arrhythmia onset.

### Cryoballoon ablation procedure

The cryoballoon ablation procedure was performed according to standard-of-care procedures, which have been previously described [3–7]. In brief, patients were sedated using general anesthesia or conscious sedation. In general, a dedicated, 15-F OD steerable sheath (FlexCath Advance Steerable Sheath; Medtronic, Inc.) was used to introduce a 28-mm cryoballoon ablation catheter (Arctic Front Advance; Medtronic, Inc.) into the left atrium (LA). The cryoballoon catheter was maneuvered in the LA either over a J-tip guidewire or a dedicated inner-lumen octopolar/decapolar circular mapping catheter (Achieve or Achieve Advance; Medtronic, Inc.). Before each ablation, the cryoballoon catheter was inflated and advanced toward the antral surface of the pulmonary vein (PV). Upon antral occlusion of the targeted PV, the cryoapplication was initiated. The number and duration of freezes per PV were determined by the physician. It was recommended to pace the right phrenic nerve during all right-sided cryoapplications to monitor phrenic nerve function during each freeze. Adjunctive imaging, intraprocedural esophageal temperature monitoring, additional ablation tools (e.g., focal cryoablation and radiofrequency catheter ablation), and/or adjunctive lesions applied to each patient were at the discretion of the operating physician. Patients were discharged from the hospital using local standard-of-care practices.

### Patient follow-up

This analysis examined patients enrolled in Japan between February 2017, and October 2018. Patients were followed according to the center’s standard-of-care protocols with a post-procedure visit required at 12 months after the index procedure. Additionally, a subset of sites continued patient follow-up until 24 months after the index procedure. Arrhythmia recurrence monitoring was not standardized by usage of a study protocol but could be conducted by any of the following methods, including electrocardiogram, Holter monitor, trans-telephonic monitor, insertable cardiac monitor, pacemaker, and/or implantable cardioverter defibrillator. Cardiovascular medications were assessed at discharge and 12 months, and general QoL was assessed by the EQ-5D-3L questionnaire at baseline and 12-month follow-up.

### Endpoints

Primary efficacy was defined as freedom from a ≥ 30-s recurrence of AF, atrial flutter (AFL) and/or, atrial tachycardia (AT) following a 90-day blanking period (during which atrial arrhythmia recurrences did not count toward the primary efficacy endpoint). The primary safety endpoint was the combined serious device- and procedure-related adverse event rate. Serious adverse events were investigator and Medtronic classified (and included all events that led to death, a serious deterioration in health, hospitalization, or a medical intervention). Arrhythmia recurrences classified by the physician as serious and device- or procedure-related were included in the primary safety endpoint for this analysis. All adverse events were followed until the event was resolved; the event was unresolved with no further actions, or the patient exited the study. Ancillary objectives included patient baseline demographics, characterization of the cryoablation procedure, and QoL after cryoballoon ablation. QoL was assessed by the EQ-5D-3L questionnaire, a generic measure of health status consisting of two parts. The first part assesses health in five dimensions (mobility, selfcare, usual activities, pain/discomfort, and anxiety/depression) each of which has three levels of response. A summary score or health state index based on the 5 dimensions ranges from 0 (least healthy) to 1 (most healthy). The second part of the questionnaire consists of a visual analog scale (EQ VAS) on which the patient rates his or her perceived health from 0 (worst) to 100 (best).

### Statistical analysis

Baseline characteristics and clinical data were summarized using the appropriate summary statistics. Continuous variables were summarized as mean and standard deviation, and categorical variables were summarized as counts and percentages. Kaplan–Meier methods were used to estimate the 12- and 24-month freedom from atrial arrhythmia recurrence and the primary safety adverse event rate. Standard error was calculated with Greenwood’s formula. EQ-5D was scored using a Japanese value set [8]. Changes in QoL from baseline to 12 months were assessed with a Student’s *t*-test. Changes in symptoms were assessed with McNemar’s test. Values of *P* < 0.05 were considered significant. Statistical analyses were conducted using the SAS software version 9.4 (SAS Institute, Cary, North Carolina).

## Results

### Patient disposition and characteristics

**Fig. 1:**
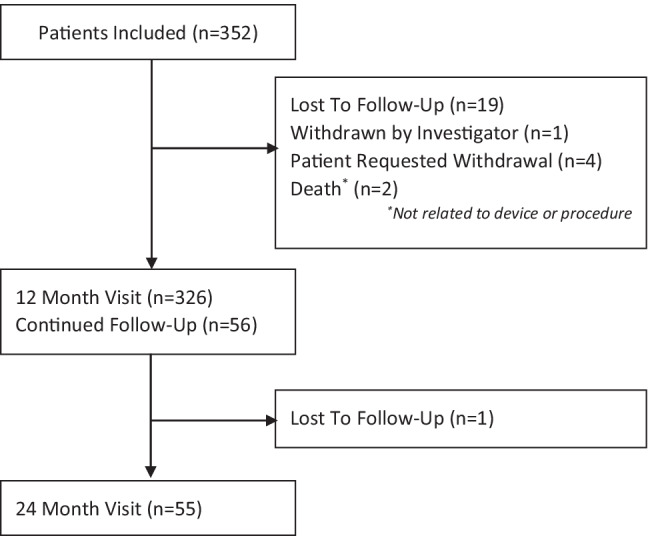
Patient flow. Patient enrollment and follow-up

Between February 2017, and October 2018, 352 eligible patients underwent a cryoballoon ablation index procedure. Expected follow-up duration was 12 (*n* = 294) or 24 months (*n* = 58) based on the enrolling center (Fig. 1). During follow-up, 25 of the 352 (7.1%) patients exited early for the following reasons, including: one patient was withdrawn by the investigator, 20 were lost to follow-up, and 4 patients requested a withdrawal. Standard of care visits before the required annual visit were performed in 268 patients for 861 visits. Baseline patient characteristics are detailed in Table 1. On average, the cohort was 65 ± 10 years of age, 35.8% female, had a CHA_2_DS_2_-VASc score of 2.2 ± 1.6, and was diagnosed with AF for a mean of 3.0 ± 5.5 (median of 0.7; *IQR* 0.3–3.4) years. All patients were diagnosed with PAF, and 36.4% of patients were deemed non-drug refractory as they had not failed a Class I or III AAD prior to the cryoablation procedure. Five patients had a prior catheter ablation for AFL (1.4%). All other patients were naïve to cardiac catheter ablation for atrial arrythmia and were undergoing an index ablation procedure.

**Table 1:** Baseline patient characteristics

Patient characteristics	(*N* = 352)
Female sex (*N* (%))	126 (35.8%)
Age in years (mean ± STD)	65 ± 10
Body mass index in kg/m^2^ (mean ± STD)	24 ± 4
Paroxysmal AF	352 (100%)
CHA_2_DS_2_-VASc score (mean ± SD)	2.2 ± 1.6
Years diagnosed with AF (mean ± STD)	3.0 ± 5.5
History of atrial flutter (*N* (%))	25 (7.1%)
History of atrial tachycardia (*N* (%))	6 (1.7%)
Left atrial diameter in mm (mean ± STD)^1^	38 ± 6
Left ventricular ejection fraction in % (mean ± STD)^2^	66 ± 8
Number of failed AADs (mean ± STD)	0.7 ± 0.7
* 0 previously failed AADs (N (%))*	128 (36.4%)
* On AAD at baseline*	8 (2.3%)
* Not on AAD at baseline*	120 (34.1%)
* 1 prior AAD failure*	158 (44.9%)
* 2 prior AAD failures*	29 (8.2%)
* 3 or more prior AAD failures*	5 (1.4%)
* Not reported*	32 (9.1%)
Hypertension (*N* (%))	188 (53.4%)
Prior cardiac device implant (*N* (%))	6 (1.7%)
*CRT-D*	2 (0.6%)
*IPG*	4 (1.1%)
Prior atrial flutter ablation	5 (1.4%)
Prior PVI	0 (0.0%)
History of congestive heart failure (*N* (%))	8 (2.3%)
NYHA classification	
* Patient does not have heart failure (N (%))*	241 (68.5%)
* Class I*	55 (15.6%)
* Class II*	3 (0.9%)
* Class III*	0 (0.0%)
* Class IV*	0 (0.0%)
* NYHA status not reported (N (%))*	53 (15.1%)
Prior myocardial infarction (*N* (%))	8 (2.3%)
Prior stroke/transient ischemic attack (*N* (%))	26 (7.4%)
Coronary artery disease (*N* (%))	14 (4.0%)
Diabetes (*N* (%))	41 (11.6%)
Sleep apnea (*N* (%))	10 (2.8%)

*AAD*, Class I and III antiarrhythmic drugs; *PVI*, pulmonary vein isolation procedure; *NYHA*, New York Heart Association classification

^1^Left atrial diameter reported in 317/352 patients

^2^Left ventricular ejection fraction reported in 341/352 patients

### Procedural characteristics

Procedure-related data are detailed in Table 2. Non-general anesthesia was utilized in 89.2% of procedures. All patients were treated with the 28-mm Arctic Front Advance (second-generation) cryoballoon. Preprocedural imaging with MRI or CT was performed in 46.3% of patients, and intraprocedural 3D electroanatomical mapping was performed in 36.4% of procedures. Phrenic nerve function was monitored during index ablation in 100% of procedures with a “pace and palpate” technique employed in 77.6% of cases. Esophageal temperature was monitored in 92.0% of the index procedures. The mean total procedure duration was 73 ± 26 min, mean LA dwell time was 45 ± 19 min, and average fluoroscopy time was 48 ± 36 min.

**Table 2:** Index procedure characteristics

Procedural characteristics	(*N* = 352)
Ablation catheter used	
* Arctic Front Advance 28 mm cryoballoon (N (%))*	352 (100%)
Mapping catheter model	
* Achieve*	301 (85.5%)
* Achieve Advance*	48 (13.6%)
Total lab occupancy time in minutes (mean ± STD)	134 ± 41
Total procedure time in minutes (mean ± STD)	73 ± 26
Left atrial dwell time in minutes (mean ± STD)	45 ± 19
Total fluoroscopy time in minutes (mean ± STD)	48 ± 36
Fluoroscopy time during cryoablation in minutes (mean ± STD)	24 ± 20
Total cryoapplication duration in minutes^7^ (mean ± STD)	15 ± 4
Sedation method (N (%))	
* General anesthesia*	38 (10.8%)
* Conscious sedation*	314 (89.2%)
Pre-procedural imaging (CT and/or MRI)	163 (46.3%)
Intra-procedural 3D electroanatomical mapping	128 (36.4%)
Intracardiac echocardiography	189 (53.7%)
Esophageal temperature monitoring (*N* (%))	324 (92.0%)
Pulmonary vein venography	348 (98.9%)
Phrenic nerve monitoring	352 (100.0%)
* Pacing / palpate*	273 (77.6%)
* Diaphragm stimulation*	127 (36.1%)
* Compound motor action potential*	267 (75.9%)
* Other*	37 (10.5%)
Pulmonary vein ablation acute success^1^ (*N* (%))	337 (95.7%)
* PVI touch-up with focal cryo catheter (N (%))*	4 (1.1%)
* PVI touch-up with focal RF catheter (N (%))*	19 (5.4%)
Isoproterenol and/or adenosine to assess PVI (*N* (%))	144 (40.9%)
Non-PVI ablation (CTI or non-CTI)	147 (41.8%)
CTI (cavotricuspid isthmus)	119 (33.8%)
Non-PVI non-CTI ablation	38 (10.8%)
* LA AF Trigger*	15 (4.3%)
* RA AF Trigger*	2 (0.6%)
* Superior vena cava vein trigger*	12 (3.4%)
* Left sided roofline*	3 (0.9%)
* Other*	9 (2.6%)
Cryoballoon applications	
*Total applications performed*	1903
*Total veins treated*	1410
* PV electrical potentials monitored (N (%))*	284 (80.7%)
* Number of applications per vein (mean* ± *STD)*	
* (mean* ± *STD)*	1.3 ± 0.7
* (median [IQR])*	1 (1, 2)
* Number of veins*	1410
* Duration of cryoapplication in seconds*	
* (mean* ± *STD)*	172 ± 44
* (median [IQR])*	180 (165, 180)
* Number of applications*	1903
* Cryoballoon nadir temperature (°C)*	
* (mean* ± *STD)*	− 48.6 ± 6.6
* (median [IQR])*	− 48 (− 54, − 44)
* Number of veins*	1410

^1^All targeted pulmonary veins isolated

Overall, 95.7% of patients had all targeted PVs acutely isolated during the index procedure. The average number of freezes per PV was 1.3 ± 0.7 for a mean duration of 172 ± 44 s. The mean balloon nadir temperature during cryoablation was − 49 ± 7 °C, and real-time PV potentials were monitored during 80.7% of cryoapplications. To complete PVI, 1.1% of patients received focal cryoablation, and 5.4% of patients received focal radiofrequency ablation. Adjunctive non-PV lesions were applied in 41.8% of patients during the index PVI procedure, including cavo-tricuspid isthmus (CTI) linear ablation in 33.8% of patients. Of the 119 patients with CTI ablation during the index procedure, 10 (8.4%) individuals had a history of atrial flutter.

### Safety

Of the 352 patients, nine serious device- or procedure-related events in nine patients occurred. The serious device- and procedure-related adverse event rate was 2.6% (95% *CI*: 1.2–4.8%). Phrenic nerve injury (PNI) occurred in seven patients, and all PNI events were ongoing at the time of hospital discharge. Of the seven total PNI events, three PNIs (43%) were symptomatic as reported by the patient, and three PNIs (43%) were classified as a serious adverse event (Table 3). Within the 12-month follow-up, six patients had resolved their PNI with no further clinical sequelae, and one patient remained unresolved. No atrioesophageal fistula, pericardial tamponade, or PV stenosis was reported over the 24-month follow-up period. A full list of serious device- or procedure-related adverse events is provided in Table 3. No deaths related to the cryoablation occurred; however, there were two deaths that were not procedure-related (due to leukemia and heatstroke) during the follow-up period.

**Table 3:** Serious device- or procedure-related adverse events

Adverse events, events (patients^#^, % patients)	Serious device- or procedure-related
Total	9 (9, 2.6%)
*Atrial fibrillation recurrence*	2 (2, 0.6%)
Phrenic nerve injury*	3 (3, 0.9%)
*Cerebral infarction*	1 (1, 0.3%)
*Hematemesis*	1 (1, 0.3%)
*Hypotension*	1 (1, 0.3%)
*Vascular pseudoaneurysm*	1 (1, 0.3%)

^#^Efficacy Analysis Cohort: total patients with an index procedure (*N* = 352)

^*^All PNI were resolved at 6 months

### Efficacy

**Fig. 2:**
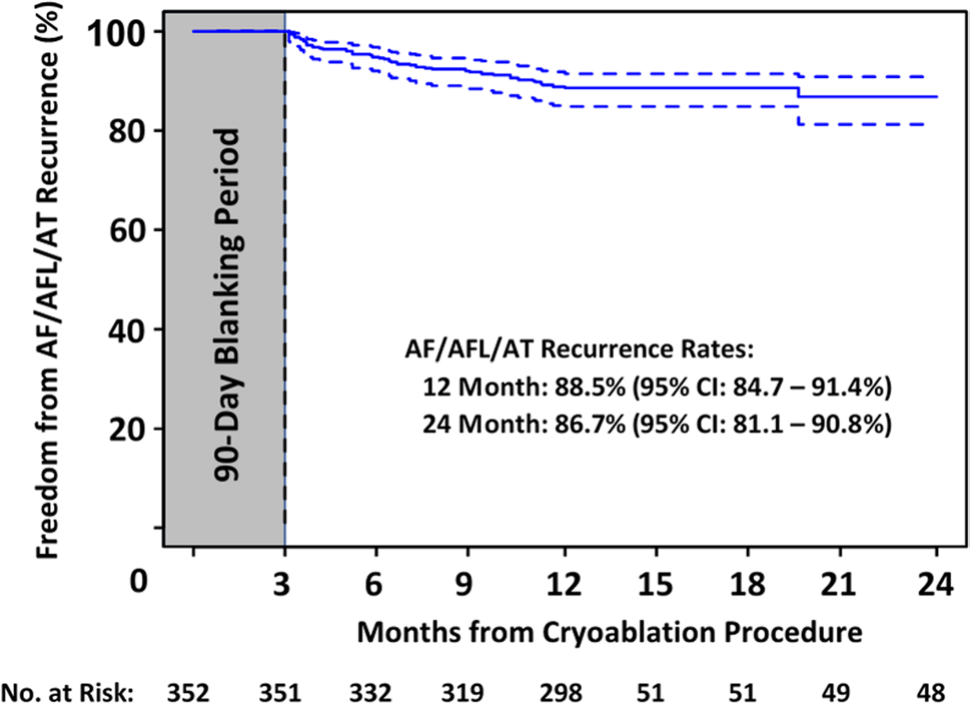
Freedom from atrial arrhythmia recurrence. Kaplan–Meier 24-month estimate of freedom from ≥ 30-s recurrences of AF/AFL/AT after a 90-day blanking period

Patients were monitored for an arrhythmia recurrence during follow-up by the following methods and frequencies, including, 12-lead ECG used in 303 (86.1%) patients during 1049 visits and Holter monitoring or more continuous monitoring used in 180 (51.1%) patients during 326 visits. A comprehensive overview of all follow-up visits with monitoring can be found in the Supplemental Table S2. The 12-month Kaplan–Meier estimate of freedom from a ≥ 30-s recurrence of AF/AFL/AT was 88.5% (95% *CI*: 84.7–91.4%) at 12 months and 86.7% (95% *CI*: 81.1–90.8%) at 24 months (Fig. 2). Irrespective of arrhythmia recurrence, the number of patients on class I/III AADs reduced from 18.5% at the index procedure discharge to 13.2% at the 12-month follow-up with further reductions at the 24-month follow-up (with 5.5% on AAD therapy). A repeat ablation was performed in 25 (7.1%) patients during the 12-month follow-up, one of which was a repeat cryoballoon ablation performed during the 90-day blanking period (Table 4). After the 12-month follow-up period, one patient received a repeat ablation, and no patient had a third catheter ablation procedure during this study period.

**Table 4:** Repeat ablations

Repeat ablations, events (patients, % patients)	Days 1–90	Days 90 +	Total
Total	1^1^ (1, 0.3%)	24^2^^,3^ (24, 6.8%)	25 (25, 7.1%)
*PVI (Cryoballoon)*	1 (1,0.3%)	0 (0%)	1 (1,0.3%)
*PVI (RF)*	0 (0%)	14 (14,4.0%)	14 (14,4.0%)
*CTI line*	1 (1,0.3%)	14 (14,4.0%)	15 (15,4.3%)
*Non-PVI left sided*	0 (0%)	8 (8,2.3%)	8 (8,2.3%)
*Non-CTI right sided*	0 (0%)	15 (15,4.3%)	15 (15,4.3%)

^1^Occurred on day 45 post-index procedure

^2^One patient reported a repeat ablation but details of what lesions were performed were not reported

^3^23 repeat ablations occurred within 1 year (days 100–359), 1 repeat ablation occurred on day 704

### AF-related symptoms and quality of life

**Fig. 3:**
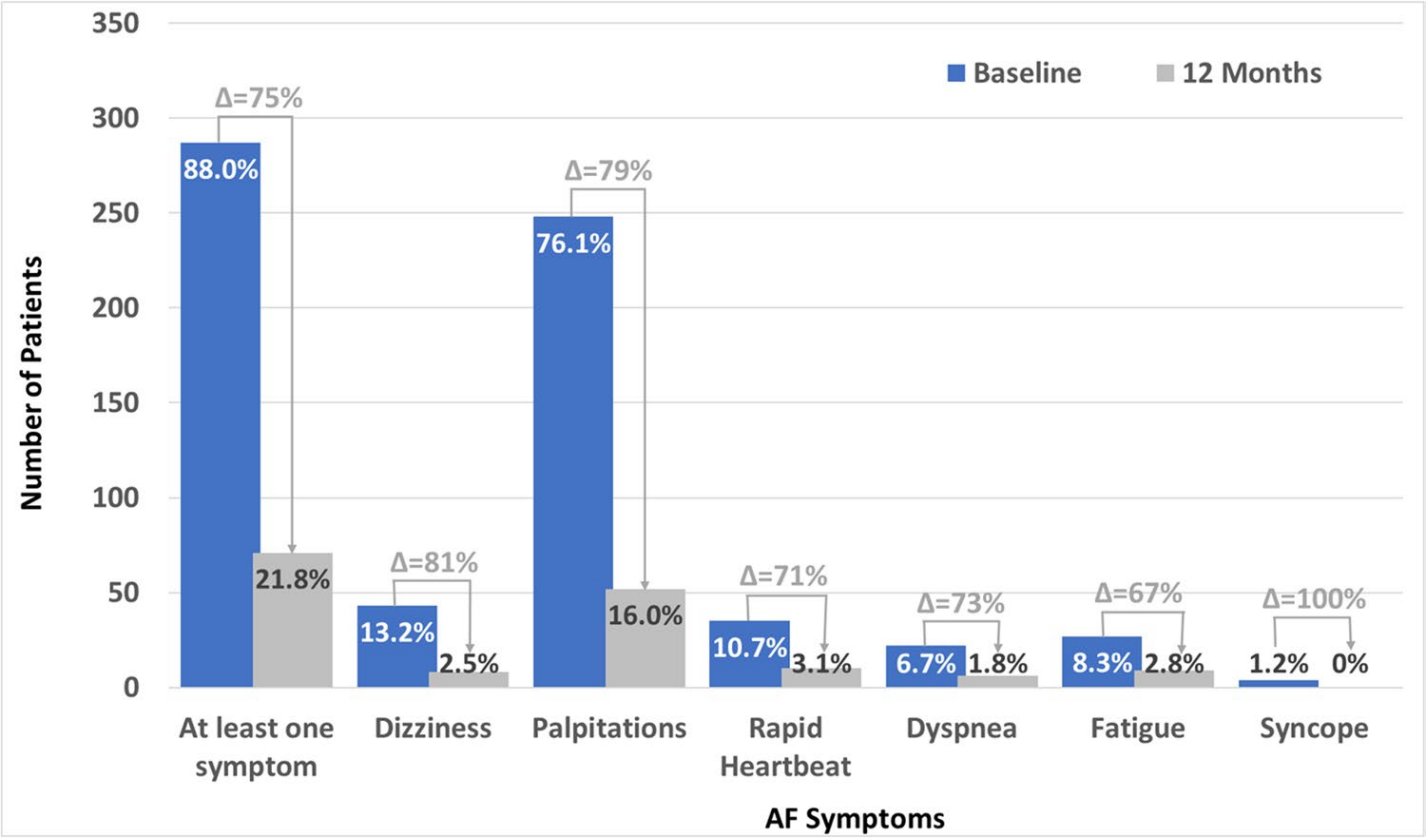
AF symptoms at baseline and 12 months. AF symptoms in 327 patients with information available during baseline and 12-month visit. ^*^Including only patients with information on symptoms during baseline and 12-month follow-up (*N* = 327)

AF-related symptoms (i.e., dizziness, palpitations, rapid heartbeat, dyspnea, fatigue, and syncope) were reported at baseline and 12-month follow-up in 326 (92.6%) patients. As shown in Fig. 3, the number of patients with ≥ 1 AF-related symptom decreased significantly from 88.0% at enrollment to 21.8% at the 12-month follow up (*p* < 0.01). Palpitations were reported most frequently at baseline with 248 (76.1%) patients; however, the number of patients with palpitation symptoms decreased to 52 (16.0%) patients at 12 months. Baseline QoL was measured by the health state index or summary score and the visual analog scale (EQ VAS) of the EQ-5D-3L. At the 12-month follow-up, there was an improvement in the summary score that was not statistically significant (0.014 ± 0.149; *p* = 0.09). However, the EQ VAS improved significantly at 12 months (5.8 ± 18.4; *p* < 0.01) as shown in Table 5.

**Table 5:** Quality of life as measured by EQ-5D-3L

	Baseline	12 months	Difference	*p*-value^2^
Health state index^3^	0.917 ± 0.141	0.931 ± 0.125	0.014 ± 0.149	0.09
Visual analog scale^4^	74.5 ± 15.8	80.3 ± 14.5	5.8 ± 18.4	< 0.01

^1^Of the 352 patients in the Efficacy Analysis Cohort, 326 completed a 12-month visit, of which 321 completed an EQ-5D questionnaire at both baseline and 12 months

^2^*t*-test

^3^Summary index score ranging from 0 to 1.00 with higher scores indicating a better health-related quality of life

^4^A vertical visual-analog scale on which patients provide a global assessment of their health, ranging from 0 to 100, with higher scores indicating a better health related quality of life

## Discussion

This analysis from the prospective, multi-center *Cryo Global Registry* evaluated the clinical performance and safety of the Arctic Front Advance cryoballoon in Japan. The serious device- and procedure-related adverse event rate was 2.6% after 24 months. Freedom from AF/AFL/AT was 88.5% at 12 months and 86.7% at 24 months. The number of patients with ≥ 1 AF symptom had significantly decreased from 88% at enrollment to 22% at the 12-month follow-up. The more general, non-AF specific, EQ-5D summary score did not improve significantly while the EQ VAS score was significantly improved by 5.8 ± 18.4 scores after 12 months. These real-world results demonstrated that cryoablation was safe and effective for the treatment of patients with PAF in Japan.

Importantly, contemporary procedural characteristics of cryoablation in Japan were observed within this registry. Adjunctive preprocedural imaging and intraprocedural mapping and monitoring were frequently performed during the cryoablation procedures. Furthermore, non-PVI lesions were commonly delivered (41.8%), with 34% of patients receiving a CTI linear ablation. History of atrial flutter was documented in 7.1% of the patients, and it is unknown whether atrial flutter was induced during electrophysiology testing in additional patients because of the limitations of data collection within this registry. Corroborating this observation, a high rate of CTI ablation was recently reported in a large retrospective study from Japan [9]. Also, PV potentials were monitored during the cryoapplication in 80.7% of patients, and on average, one cryoapplication was delivered to each PV for a median of 180 s. This observation indicates a shift in dosing strategy since the CRYO-Japan PMS study in which two applications were delivered per PV for approximately 150 s on average [1]. Since the PMS study, procedural best practices have been established to optimize PVI delivered by the Arctic Front Advance system through cryoballoon positioning techniques and freeze-tailored dosing based on PV potential monitoring [7, 10, 11]. Furthermore, procedure times were short at 73 min (compared to 150 min in the Japan PMS study). The short procedure times observed in the Japan Cryo Registry may be related to increased usage of tailored dosing paradigms and increased operator experience (since the cryoballoon catheter was first introduced in Japan). Additionally, the low AF/AFL/AT recurrence rate of 1.8% starting from 12 until 24 months compared to the 11.5% recurrence from end of the blanking period until the 12-month follow-up demonstrates an excellent longer-term lesion durability of the Arctic Front Advance cryoballoon using procedural best practices in Japan.

The procedure- and device-related serious adverse event rate was low in this study (2.6% after 24 months). While PNI was the most frequently observed adverse event in this cohort, the total number of serious and non-serious PNI events (7 in total) and the single PNI event ongoing at 12 months (0.3%) are in agreement with other recent studies [3, 9, 12]. Moreover, the rate of PNI in this current study is lower than older studies using the first-generation cryoballoon [4]. The short-term, 6-month efficacy of Arctic Front Advance cryoballoon ablation (for the treatment of patients with PAF conducted according to real-world practice across 33 centers after the initial approval of the cryoablation catheter in Japan in 2014) was 88.4% [1]. In this registry, the real-world freedom from atrial arrhythmia reoccurrence at 24 months was 86.7% with a low rate of repeat ablation over the follow-up period. These 24-month results are similar to the efficacy in patients with PAF in other contemporary registries that have followed patients after typical clinical practices in Europe and the USA [13–16]. Furthermore, AF-related symptoms were significantly reduced by 75% between baseline and 12 months with only 22% of patients reporting AF-related symptoms at 12 months. QoL (as measured by the summary score of the EQ-5D-3L) was high at baseline and a non-significant trend in improvement was observed at 12 months; however, the respondents self-rated health measured by the EQ VAS reporting improved significantly. As symptom reduction is a primary objective in this patient population [17–19], these results support the utility of cryoablation for patients with symptomatic PAF in Japan.

## Limitations

This sub-analysis of the observational *Cryo Global Registry* describes the clinical performance and safety of patients treated with Arctic Front Advance in Japan according to real-world clinical usage. Arrhythmia monitoring was performed to the standard-of-care methods at each site. At 12 months follow-up, data for monitoring beyond ECG was only available in 1/3 of the patients; however, at 24 months follow-up, data for monitoring was present in nearly 2/3 of the patients. Therefore, episodes of asymptomatic AF might not have been uniformly identified, but the longer-term efficacy data is supported by vigilant arrhythmia monitoring of patients. QoL with the EQ-5D summary score at baseline in this cohort was 0.917 (with a maximal score of 1). Consequently, improvements in QoL may not have been detectable with the EQ-5D-3L summary score (a more generalized tool) in this cohort. Finally, not all patients were followed up for > 12 months as an initial protocol, and only a limited number of patients were studied for a longer-term outcome.

## Conclusions

This study demonstrates that PVI with the Arctic Front Advance cryoballoon catheter is a safe and effective treatment in the real-world clinical usage for patients suffering from symptomatic PAF in Japan.

## Supplementary Information

Below is the link to the electronic supplementary material.

Supplementary file1 (PDF 169 KB)

## Abbreviations

AAD: Antiarrhythmic drug
AF: Atrial fibrillation
AFL: Atrial flutter
AT: Atrial tachycardia
CTI: Cavotricuspid isthmus
EQ-5D: EuroQol-5 Dimension
EQ VAS: EQ-5D visual analog scale
PAF: Paroxysmal atrial fibrillation
PNI: Phrenic nerve injury
PV: Pulmonary vein
PVI: Pulmonary vein isolation
QoL: Quality of life
RF: Radiofrequency
