# Supplementary material for: Cryoballoon ablation for paroxysmal atrial fibrillation in Japan: 2-year safety and efficacy results from the Cryo AF Global Registry
Source: J Interv Card Electrophysiol. 2022 Feb 4;64(3):695–703. doi: 10.1007/s10840-022-01132-0 (PMC9470593; doi:10.1007/s10840-022-01132-0)
Supplement: Supplementary file 1 — Supplementary file1 (PDF 169 KB) [file 10840_2022_1132_MOESM1_ESM.pdf]

Supplemental Table S1. Enrollments per Site

A total of 10 sites in Japan participated in this data analysis.

Prefecture, Country	Investigator	Site	Enrolled Patients
Kumamoto, Japan	Prof. Ken Okumura	Saiseikai Kumamoto Hospital	30
Fukuoka, Japan	Dr. Kenji Ando	Kokura Kinen Hospital	30
Kyoto, Japan	Dr. Satoshi Shizuta	Kyoto University Hospital	40
Hyogo, Japan	Dr. Atsushi Kobori	Kobe City Medical Center General Hospital*	30
Kanagawa, Japan	Dr. Yasuteru Yamauchi	Yokohama City Minato Red Cross Hospital	38
Aomori, Japan	Dr. Masaomi Kimura	Hirosaki University Hospital*	28
Saitama, Japan	Dr. Osamu Inaba	Saitama Red Cross Hospital	40
Osaka, Japan	Prof. Takashi Kurita	Kindai University Hospital	40
Tokyo, Japan	Prof. Masahiko Goya	Tokyo Medical and Dental University, Medical Hospital	40
Fukuoka, Japan	Dr. Koichiro Kumagai	Fukuoka Sanno Hospital	36

*site continued follow-up after 12 months

Supplemental Table S2. AF Monitoring per Follow-up Visit

Described is the number of visits and patients with the arrhythmia monitoring used. A total of 352 patients were enrolled and treated with cryoballoon ablation. Post-ablation during follow-up, 303 (86.1%) patients received arrhythmia monitoring by 12-lead ECG and 180 (51.1%) received arrhythmia monitoring by Holter or remote monitoring. In two (0.6%) patients implanted cardiac devices with continuous arrhythmia monitoring where used, and 7 (2.0%) patients received external event monitors.

	Study Visits (Patients)					
	**Baseline (352 Pts)**	**Unscheduled Baseline-12M (268 Pts[1])**	**12-Month (326 Pts)**	**Unscheduled 12-24 Month (0 Pts)**	**24-Month (55 Pts)**	**Total (352 Pts)**
**12-lead ECG**	311 (88.4%)	772 (95.9%)	233 (71.5%)	0	44 (80.0%)	1049 (86.1%)
**Holter/Remote Monitor**	57 (16.2%)	180 (44.8%)	109 (33.4%)	0	37 (67.3%)	326 (51.1%)
Holter ≤ 24h	10 (2.8%)	157 (41.4%)	101 (31.0%)	0	37 (67.3%)	295 (47.4%)
Holter > 24h	1 (0.3%)	4 (1.5%)	2 (0.6%)	0	0	6 (1.4%)
Holter length unknown	0 (0%)	1 (0.4%)	2 (0.6%)	0	0	3 (0.9%)
Implanted Event Monitor[2]	1 (0.3%)	12 (0.7%)	2 (0.6%)	0	0	14 (0.6%)
External Continuous Event Monitor	11 (3.1%)	0 (0%)	1 (0.3%)	0	0	1 (0.3%)
External Event Monitor	34 (9.7%)	7 (1.9%)	2 (0.6%)	0	0	9 (2.0%)

[1] 861 unscheduled visits occurred between Baseline and 12 months.

[2] Arrhythmia monitoring from an implanted cardiac device reviewed (eg. IPG, ICD, CRT, ILR)
